# Swedish Mechanical Exercise

**Published:** 1889-03-02

**Authors:** 


### SWEDISH MECHANICAL EXERCISE.

The Zander mechanical exercises are chiefly intended for those whose age and physical condition are unequal to the strain of ordinary exercises, as "ladies, young girls, elderly men, and weakly persons of all ages." The different parts of the body are exercised by machinery, so that a person may sit at ease while his legs and arms are exercised without any expenditure of physical force. There is also a friction machine for the feet, and a percussion machine for any part of the body. Medical electricity is also added to the system. There is no doubt that both passive exercises and electricity are occasionally of great utility in various forms of disease, and probably a combination of the two would some times prove still more effectual. We are told that cases of spinal curvature, rheumatism, neuralgia, sciatica, lumbago, partial paralysis, constipation, feeble circulation, stiff joints, contracted limbs, diabetes mellitus, pulmonary tendencies, etc., are all to be cured or benefited by this treatment. In this age of progress it is not well to deny anything; but, notwithstanding all we are told, we fear that the promoters of this system, after the manner of specialists generally, "do protest too much" for their specialitè.

